# TRENDS IN OPIOID USE IN LONG-TERM CARE NURSING HOME RESIDENTS WITH DEMENTIA

**DOI:** 10.1093/geroni/igz038.2605

**Published:** 2019-11-08

**Authors:** Hemalkumar B Mehta, Yong-Fang Kuo, Jordan Westra, Mukaila Raji, James S Goodwin

**Affiliations:** The University of Texas Medical Branch, Galveston, Texas, United States

## Abstract

We examined opioid use in long-term care nursing home residents with dementia. This retrospective cohort study used Minimum Data Set linked Medicare data, 2011-2016, and included long-term care episodes for residents 65+ years who survived 100+ days each year (592,211 episodes for 256,207 residents). Cognitive status at first annual assessment was classified as none/mild, moderate and severe impairment. Overall opioid use, prolonged opioid use (prescription supply 90+ days) and long-acting opioid use were identified from Medicare part D. Descriptive statistics were used to describe opioid use by cognitive impairment. Cochrane Armitage trends test was used to determine trends in opioid use. 114,622 (19%) patients had severe and 129,257 (22%) had moderate dementia. Overall opioid (none/mild=15.4%, moderate=13.9%, severe=9%), prolonged opioid (none/mild=5.2%, moderate=4.5%, severe=3.2%) and long-acting opioid use (none/mild=1.1%, moderate=0.9%, severe=0.3% ) were lower in patients with advanced dementia. Opioid use was significantly higher in females and Whites and varied by states. Substantial increase was found in overall opioid and prolonged opioid use from 2011 to 2016, with greater increase in none/mild and moderate dementia patients. For example, prolonged opioid use increased by 69% in none/mild and 71% in moderate dementia patients compared to 52% in severe dementia patients (p<0.0001). Long-acting opioid use decreased, with a greater decline in none/mild (69%) and moderate (71%) dementia patients compared to severe dementia patients (58%) (p<0.0001). Contrary to decreasing opioid use in community setting, overall and prolonged opioid use increased in nursing home residents. Future studies should identify the reasons behind increased use.

